# Roles of calcium in ameloblasts during tooth development: A scoping review

**DOI:** 10.1016/j.jtumed.2024.12.010

**Published:** 2024-12-30

**Authors:** Islamy R. Hutami, Dian Y. Arinawati, Arief Rahadian, Rizqa C. Dewi, Yayun S. Rochmah, Sandy Christiono, Shaista Afroz

**Affiliations:** a Department of Orthodontics, Faculty of Dentistry, Universitas Islam Sultan Agung, Indonesia; b Department of Oral Biology, Faculty of Dentistry, Universitas Muhammadiyah Yogyakarta, Indonesia; c Department of Biochemical, Faculty of Medicine, Universitas Islam Sultan Agung, Indonesia; d Master Program of Dental Sciences, Faculty of Dentistry, Universitas Islam Sultan Agung, Indonesia; e Department of Oral Surgery, Faculty of Dentistry, Universitas Islam Sultan Agung, Indonesia; f Department of Pediatric Dentistry, Faculty of Dentistry, Universitas Islam Sultan Agung, Indonesia; g Department of Prosthodontics/Dental Material, Dr. Ziauddin Ahmad Dental College, Aligarh Muslim University, India

**Keywords:** Ameloblast, Amelogenesis, Calcium, Enamel, Tooth development, المينا, خلايا المينا, تكوين المينا, الكالسيوم, تطور الأسنان

## Abstract

**Objectives:**

Calcium ions (Ca^2+^) play crucial role in tooth development, particularly in maintaining enamel density during amelogenesis. Ameloblasts require specific proteins such as amelogenin, ameloblastin, enamelin, kallikrein, and collagen for enamel growth. Recent research has highlighted the importance of calcium and fluoride ions, as well as the TRPM7, STIM, and SOCE pathways, in regulating various stages of enamel formation. This review synthesizes current knowledge, focusing on preclinical data elucidating the molecular mechanisms of calcium transport in ameloblasts, during normal tooth development and in response to external stimuli.

**Methods:**

This scoping review followed the Preferred Reporting Items for Systematic Reviews and Meta-Analyses (PRISMA) guidelines. The literature search, conducted in December 2023, spanned multiple databases including PubMed (8.363 records), Google Scholar (5.630 records), and Science Direct (21.810 records). The primary aim was to examine the influence of calcium ion regulation on ameloblast development, with a focus on preclinical studies.

**Results:**

After an initial screening of 396 titles and abstracts, 11 full-text articles (four *in vitro* studies and seven animal studies) met the inclusion and exclusion criteria. The studies, assessed for quality using the CAMRADES tool, ranged from low to moderate. Calcium deficiency, nutritional supplements, fluoride exposure, TRPM7, STIM proteins, and the SOCE pathway were found to influence amelogenesis.

**Conclusion:**

Calcium transport mechanisms play a critical role in enamel formation, with factors such as TRPM7, Kir 4.2, CRAC channels, and the SOCE pathway supporting enamel mineralization, while disruptions like hypoxia, fluoride exposure, and circadian imbalances negatively impact amelogenesis. Understanding the interplay between calcium, environmental, and nutritional factors provides valuable insights into ameloblast function and offers potential avenues for improving enamel quality and addressing defects.

## الملخص

**أهداف البحث:**

تلعب أيونات الكالسيوم دورا حيويا في تطور الأسنان، خاصة في الحفاظ على كثافة المينا أثناء تكوينها. تحتاج خلايا المينا إلى بروتينات محددة مثل الأميلوجينين والأميلوبلاستين والإيناملين والكاليكرين والكولاجين لنمو المينا. يسلط البحث الحديث الضوء على أهمية أيونات الكالسيوم والفلورايد، إلى جانب مسارات مختلفة، في تنظيم مراحل تكوين المينا. تهدف هذه المراجعة إلى تجميع المعرفة الحالية، مع التركيز على البيانات قبل السريرية لتوضيح الآليات الجزيئية لنقل الكالسيوم في خلايا المينا.

**طرق البحث:**

اتبعت هذه المراجعة إرشادات بريزما. أجري البحث في ديسمبر 2023 في قواعد بيانات متعددة شملت بابميد (8,363 سجل)، وجوجل سكولار (5,630 سجل)، وساينس دايركت (21,810 سجل). كان الهدف الرئيسي دراسة تأثير تنظيم أيونات الكالسيوم على تطور خلايا المينا، مع التركيز على الدراسات قبل السريرية.

**النتائج:**

بعد فحص أولي لـ 396 عنوانا وملخصا، استوفت 11 مقالة كاملة (أربع دراسات مخبرية وسبع دراسات حيوانية) معايير الإدراج والاستبعاد. تراوحت جودة الدراسات، التي تم تقييمها باستخدام أداة كامراديس، من منخفضة إلى متوسطة. أشارت البيانات إلى أن نقص الكالسيوم، والمكملات الغذائية، والتعرض للفلورايد، والبروتينات المختلفة، والمسارات الخلوية تؤثر على تكوين المينا.

**الاستنتاجات:**

كشفت الأبحاث باستخدام النماذج ثلاثية الأبعاد، وتحليل التعبير الجيني، والحيوانات المعدلة وراثيا عن تفاعل معقد بين أيونات الكالسيوم والعوامل البيئية والغذائية التي تؤثر على وظيفة خلايا المينا وتطور المينا. تتضمن العوامل المعززة لتمعدن المينا قنوات مختلفة ونقل الكالسيوم عبر مسارات متعددة والكالسيوم الغذائي. في المقابل، تؤثر زيادة المنشطات اليومية، ونقص المثبطات اليومية، ونقص الأكسجين، والتعرض للفلورايد سلبا على تكوين المينا.

## Background

Calcium ions (Ca^2+^) are indispensable in tooth development, whereas calcium deficiency can damage hard dental tissues, including the enamel and dentin.^1^ Abnormalities such as enamel hypoplasia and hypomineralization manifest as pits and lines on the tooth enamel surface, and make the teeth susceptible to caries, fracture, and craze line development after eruption.^2^

Amelogenin is a protein involved in enamel development. Ameloblasts play crucial roles in enamel development, from secretion to post-secretory stages.^2^^,^^3^ Secretory ameloblasts provide an organic protein matrix for enamel crystal elongation; mature ameloblasts facilitate ion transport and are involved in protein removal, thereby enabling the crystals to gain width and thickness.^4^ Recent studies of ameloblast physiology have indicated that Ca^2+^ absorption into ameloblasts is regulated by Ca^2+^ entry pathways that operate with storage mechanisms.^5^^,^^6^

New developments in dental research have yielded a wide range of findings that have advanced understanding of the complexities of amelogenesis.^7^ For example, next-generation sequencing has elucidated the molecular mechanisms underlying amelogenesis disorders and enabled identification of the genetic variations causing amelogenesis imperfecta, such as WD repeat domain (WDR)72 and stromal interaction molecule (STIM)1.^8^, ^9^, ^10^ One notable breakthrough has been the establishment of a three-dimensional (3D) model providing a representative and dynamic platform for the study of ameloblast behavior.^11^^,^^12^ This innovative model has facilitated in-depth understanding of the molecular mechanisms underlying enamel formation and the pathophysiology of enamel-related disorders.

Enamel mineralization is a highly regulated process requiring precise control of Ca^2+^ transport to support the formation and maturation of this uniquely hard tissue. During enamel formation, Ca^2+^ transport mechanisms provide a steady supply of ions to the mineralizing front, thus facilitating the growth of hydroxyapatite crystals, which serve as a structural foundation. Ca^2+^ channels, pumps, and transporters play critical roles in maintaining calcium homeostasis, whereas disruptions in their pathways can lead to enamel defects.^13^, ^14^, ^15^ Recent studies have highlighted the roles of specific Ca^2+^ channels in the modulation of Ca^2+^ levels in ameloblasts, which adapt to high Ca^2+^ demand during enamel secretion and maturation.^1^^,^^16^ However, the specific pathways and proteins mediating Ca^2+^ transport in ameloblasts during different stages of enamel formation remain incompletely understood.

To further elucidate these mechanisms, the contributions of transient receptor potential cation channel subfamily M member 7 (TRPM7)^2^^,^^17^^,^^18^ and store-operated calcium entry (SOCE) in ameloblasts must be explored.^16^^,^^18^^,^^19^ The identification of the TRPM7 and SOCE pathways, which play essential roles in the regulation of Ca^2+^ influx, has substantially advanced understanding of Ca^2+^ transport in ameloblasts.^2^^,^^17^^,^^18^^,^^20^ Dysregulation of either of these pathways can lead to enamel defects, but their crosstalk suggests that targeting both mechanisms might enhance the effectiveness of amelogenesis disorder treatments.

Energy metabolism has emerged as another critical aspect of enamel formation.^21^ Under hypoxic conditions, a metabolic shift in ameloblasts affects their maturation and prompts the differentiation of ruffle-ended ameloblasts into smooth-ended ameloblasts. This phenotypic alteration affects enamel development, and its identification has provided novel insights into the metabolic underpinnings of amelogenesis.^22^ Furthermore, recent studies have revealed notable interactions among Ca^2+^, fluoride ions (F⁻), and ameloblast activity.^24^^,^^25^ These findings suggest the feasibility of potential approaches to prevent fluorosis—a condition associated with excessive fluoride exposure—and highlight strategies to safeguard dental health against environmental factors.^23^^,^^24^

Complementing these insights, research has shown that Kir4.2 plays roles in sodium ion (Na^+^) and potassium ion (K^+^) absorption, as well as pH-dependent ion uptake regulation, during enamel maturation.^23^, ^24^, ^25^, ^26^ These findings have expanded understanding of ion transport mechanisms and revealed potential therapeutic targets for the optimization of enamel health. Together, these diverse research findings have led to substantial progress in dental research. Herein, a comprehensive analysis of evidence from preclinical studies is presented, to elucidate the molecular mechanisms of Ca^2+^ transport and their effects on ameloblast function throughout various stages of tooth development, under physiological conditions and in response to extrinsic factors.

## Materials and Methods

### Design and search methods

We applied the scoping review method for transparent and systematic collection of diverse data, and the generation of a detailed summary of findings.^27^^,^^28^ This approach aids in understanding of the advantages and limitations of exploratory investigation.^29^ The review was conducted as described by Arskey and O'Malley.^30^

The PubMed, Google Scholar, and Science Direct databases were searched for relevant articles to obtain secondary data obtained by other researchers. The systematic electronic search was performed in a stepwise manner. The specific keywords used in the databases were “calcium OR calcium ion OR atomic number 20 OR calx OR calcium oxide OR lime OR factor IV OR quicklime OR burnt lime OR calcined lime OR Ca OR fluxing lime AND ameloblast OR cell-free AND mouth OR rima oris OR oral cavity OR tooth OR teeth OR primary dentition OR secondary dentition OR dentition OR oral fissure AND growth OR development OR life cycle OR maturation OR biological process OR growing OR teething OR ontogenesis.” Duplicate articles obtained after keyword searches in the databases were removed, and the remaining articles’ titles and abstracts were screened. Subsequently, full-text articles were analyzed for eligibility according to the inclusion/exclusion criteria. Two authors independently performed all stages of the analysis, and any disagreements were resolved by discussion. Pertinent information from the included articles was compiled with a specialized template. The review was conducted in accordance with the Preferred Reporting Items for Systematic Reviews and Meta-Analyses for Scoping Reviews (PRISMA ScR) guidelines in December of 2023.

### Inclusion and exclusion criteria

The population, concept, and context of the included studies were ameloblasts; effects of Ca^2+^ on ameloblasts during proliferation, differentiation, and maturation; and tooth development. Research articles published in the past 5 years (2018–2023) for which full texts were accessible were included. Reports on animal and *in vitro* studies were included. Research articles not in English or abstracts were excluded.

### Quality assessment and data extraction

The quality of the included studies was assessed with the Collaborative Approach to Meta-analysis and Review of Animal Data from Experimental Studies (CAMRADES) guidelines^31^ (checklist in Table 1). If the answer to a question was yes (Y), a score of 1 was assigned. If the relevant information could not be procured, no score was given. For animal studies, 12 items were assessed; for *in vitro* studies, nine items were assessed. Higher scores were considered to reflect better study quality. Data extracted from the included studies were compiled in tabular format according to the problem/population, intervention, control/comparison, outcome framework.^32^^,^^33^ The extracted information comprised the authors, year of publication, study purpose, population, experimental group intervention, control group conditions, outcomes and measures, results, and clinical implications of the findings.^31^

**Table 1:** Critical appraisal of included studies with the CAMRADES quality assessment tool.

	Kádár K et al., 2021^2^	Said et al., 2020^5^	Arai et al., 2022^21^	Ngu et al., 2023^65^	Christiono et al. 2021 ^3^	Földes et al., 2021^11^	Liu et al., 2021^4^	Costiniti et al., 2022^16^	Christiono et al., 2022^52^	Gao et al., 2020^40^	Nurbaeva et al., 2018^19^
Study type	Cell culture	Animal	Animal	Animal	Animal	Cell culture	Cell culture	Animal	Animal	Cell culture	Animal
Q1	Y	Y	Y	Y	Y	Y	Y	Y	Y	Y	Y
Q2	Y	Y	Y	Y	Y	Y	Y	Y	Y	Y	Y
Q3	N	N	N	N	N	N	N	N	N	N	N
Q4	N	N	N	N	N	N	N	N	N	N	N
Q5	N	N	N	N	N	N	N	N	N	N	N
Q6	Y	Y	Y	Y	N	Y	Y	Y	Y	Y	Y
Q7	N	N	N	N	Y	N	N	N	N	N	N
Q8	Y	Y	Y	N	N	Y	Y	Y	Y	Y	Y
Q9	N	Y	Y	Y	Y	N	N	Y	Y	Y	Y
Q10	NA	Y	N	N	N	NA	NA	N	N	NA	N
Q11	NA	N	N	N	Y	NA	NA	N	N	NA	N
Q12	NA	N	N	N	Y	NA	NA	N	N	NA	N
Score	4/9	6/12	5/12	4/12	6/12	4/9	4/9	5/12	5/12	5/9	5/12
RoB	High	Moderate	High	High	Moderate	High	High	High	High	Moderate	High

Criteria of the question (Q): Q1, Peer reviewed publication; Q2, Control used for outcome assessment; Q3, Allocation concealment; Q4, Blinded assessment of outcome; Q5, Sample size calculation; Q6, Statement of conflict of interest; Q7, Prespecified inclusion exclusion criteria; Q8, Reporting of study funding; Q9, Statement of compliance with regulatory requirements; Q10, Statement of control of temperature; Q11, Reporting of animals excluded from analysis; Q12, Randomization to treatment and control. Q 10, 11, and 12 are only for animal studies (yes: Y; no: N).

## Results

**Figure 1:**
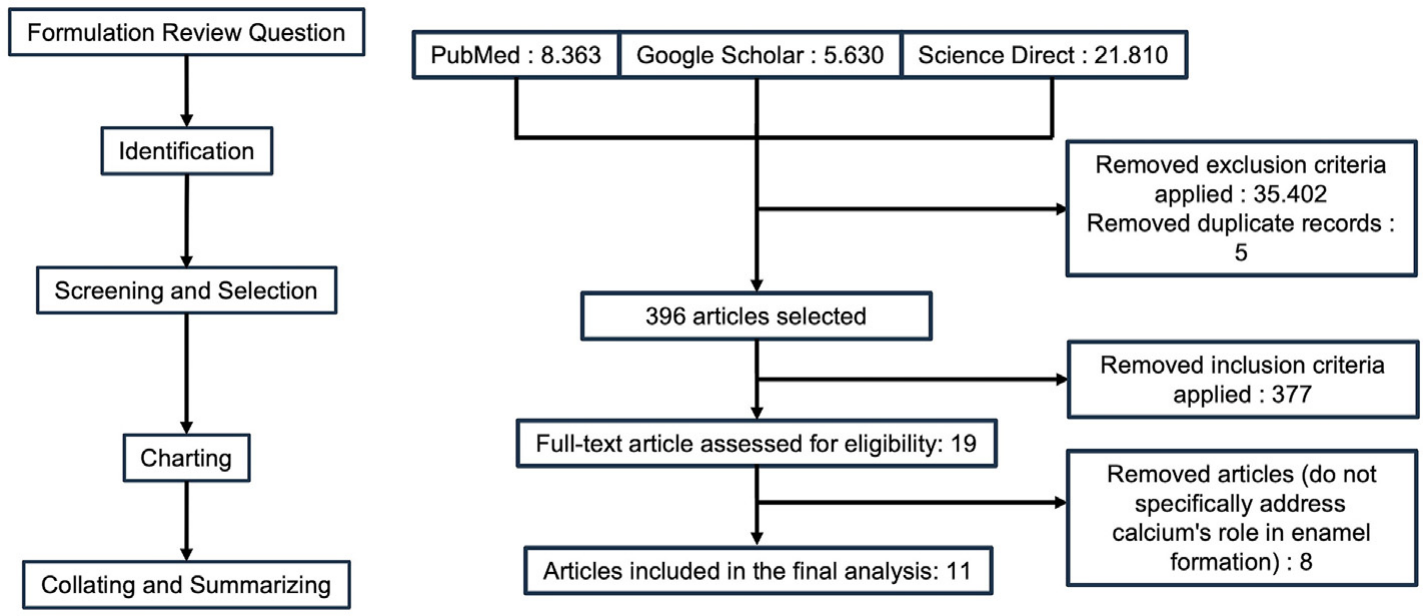
**Preferred Reporting Items for Scoping Review diagram**. The initial electronic search yielded 35,803 potentially eligible articles, and no additional studies were identified through a manual search. Rigorous selection, including the application of exclusion criteria and removal of duplicates, led to the identification of 396 distinct articles. Application of the inclusion criteria to these records yielded 19 relevant articles. Eight of these articles were excluded because they did not specifically address calcium's role in enamel formation. Ultimately, 11 articles were selected and analyzed, thus providing a focused and comprehensive examination of calcium's function in enamel development.

The PubMed, Google Scholar, and Science Direct searches yielded 8,363, 5,630, and 21,810 article records, respectively. Preliminary screening of the titles and abstracts and duplicate removal yielded 396 unique articles. Of these, 11 articles were deemed eligible for full-text evaluation. The flow of data retrieval is illustrated in Figure 1.

All included studies were of moderate to low quality (Table 1). All articles were peer-reviewed publications describing studies in which control groups were used for outcome assessment. One article did not include a conflict of interest statement; funding was not reported in two articles; and compliance with regulatory requirements was not reported in three articles. No studies involved allocation concealment, blinded outcome assessment, or sample size calculation. The application of prespecified inclusion and exclusion criteria was reported in one article. One of the seven articles on animal studies reported temperature control, exclusion of animals from the analysis, and randomization to treatment and control groups.

Comprehensive information regarding the included studies is provided in Table 2. The most frequently used ameloblasts were the HAT-7 line (three studies), LS8 line (two studies), and ameloblast-lineage cells (ALCs; one study). The animal studies were conducted in rats and mice, including genetically modified [Wdr72^−/−^, Ncks4^−/−^, and STIM conditional knockout (cKO)] mice.

**Table 2:** Characteristics of the included studies.

Reference	Purpose	Population	Intervention	Control	Outcome measurement tools	Outcome measured	Main result	Clinical implications
Kádár et al., 2021^2^	Analysis of calcium transport facilitated by TRPM7 channels in amelogenesis	HAT-7 ameloblasts	TRPM7 inhibitors: NS8593 and FTY720; TRPM7 activators: Naltriben and mibefradil; SOCE inhibitor: BTP2	Internal control for RT-qPCR: Acidic ribosomal protein P0; negative control for IHC: Non specific Rabbit IgG	RT-qPCR, IHC, electrophysiology, Ca^2+^ imaging, intracellular pH measurement	Expression of TRPM7 channels, Ca^2+^ influx in the presence of TRPM7 activator and inhibitor	High expression of TRPM7 channels serves as a Ca^2+^ uptake pathway and is sensitive to pH changes	Enamel mineralization
Said et al., 2020^5^	Analysis of correlation between Ca^2+^ and circadian cycles in tooth enamel formation	Mice	Deletion of the Stim1 gene in ameloblasts (Stim1fl/fl/Amelx-iCre^+/+^, Stim1 cKO)	Stim1fl/fl/Amelx-iCre^−/−^)	PCR array, qRT-PCR, IHC	Circadian clock signaling genes and proteins in ameloblasts after Stim 1 deletion	Increased expression of circadian activator gene Bmal1, decreased expression of the circadian inhibitor gene period 2 (Per2)	Developmental defect in amelogenesis
Arai et al., 2022^21^	Examination of changes in energy metabolism influencing the development of ameloblast phenotypes at various stages of maturation	Ddy mice (immunostaining), Wistar rats (ameloblasts for cytochrome oxidase activity), and HAT-7 ameloblast cell line	Cell cultures under normoxic conditions (5% CO_2_ and 21% O_2_) and hypoxic conditions (5% CO_2_, 5% O_2_, and 90% N_2_)	Positive control for apoptosis induction: Mitomycin C treated cells	IHC, IF, RT-PCR, transmission electron microscopy	Expression pattern of energy metabolic enzymes in mature ameloblasts	Hypoxia induced glycolytic dominant state, causing decreased alkaline phosphatase, and calcium transport and deposition; phenotypic shift from ruffle-ended to smooth-ended ameloblasts	Pathogenesis of enamel hypomineralization
Ngu et al., 2023^65^	Examination of transport of Na^+^ and K^+^ ions, as well as regulation of ameloblast cell development	Nckx4^−/−^, Wdr72^−/−^ C57BL/6 WT mouse lines, Postnatal 40-day mice, LS8 cells	0 or 50 ppm fluoride in drinking water for 5 weeks	Drinking water without fluoride; kidneys for IHC and WB	RNA-seq analysis, WB, IHC, RT-qPCR	Expression of K^+^ exchangers and channels in secretory and maturation stages of enamel organs	Kir4.2 (Kcnj15) mediated inward K^+^ flux in maturation ameloblasts	Pathogenesis of fluorosis in enamel
Christiono et al. 2021^3^	Examination of effects of seawater fish nanoparticles in the maternal diet on the density of tooth enamel in offspring	Pregnant female mice (*Mus musculus*) and their pups	Saltwater fish nanoparticle powder in pregnant female mice	Administration of distilled water	CBCT or μ-CT	Enamel density	Higher enamel density in the treatment group	Maternal diet influences dental health in offspring
Földes et al., 2021^11^	Enhancement of the culture conditions for the three-dimensional growth of HAT7 cells and investigation of the effects of fluoride exposure on the production of HAT7 spheroids	HAT7 cells	Extracellular matrix in three distinct growth conditions and medium containing various concentrations of fluoride	Cell culture in control medium	RT-qPCR, microfluorometry, phase contrast light microscopy	Expression of ion transporter and tight junction proteins, intracellular calcium, pH levels	3D multicellular, spherical formations of HAT7 cells with ability to regulate pH and facilitate intracellular Ca^2+^ signaling; fluoride in the culture medium adversely affects the morphology of spheroids in a dose and time dependent manner	3D model: Amelogenesis, in health and disease, for studying the concentration dependent Damaging effects of fluoride on amelogenesis
Liu et al., 2021^4^	Exploration of the effects and mechanisms of Ca^2+^ supplementation on fluoride in the ALC pathway	Murine ALC	Ca^2+^ supplementation	No calcium supplementation	qRT-PCR, Laser confocal microscopy, flow cytometry, WB	Effects of fluoride and various Ca^2+^ levels on proliferative activity, cell apoptosis, and cell cycle	Fluoride-induced apoptosis and KLK4 inhibition reversed by Ca^2+^; fluoride-induced ER stress pathway is decreased by Ca^2+^ supplementation	Ca^2+^ supplementation antagonizes fluorosis
Costiniti et al., 2022^16^	Examination of the effects of mitochondria on calcium signaling in ameloblasts	SD male rats for primary enamel cell culture, murine LS8 cells	Mandibular incisor teeth of mice (collected, cultured, and subsequently examined)	1 μM FCCP treatment as a control for mitochondrial depolarization; lysates of HEK-293 cells negative control for WB	RT-PCR, Eclipse microscopy, Flexstation 3, spectrometry, WB	Quantification of cCa^2+^ and mCa^2+^, mitochondrial depolarization, effects of MCU blocker in enamel cells	Mitochondria aid in enamel mineralization by supplying high levels of ATP and differentially buffering Ca^2+^ fluxes via SOCE	Physiological role of mitochondria in enamel mineralization
Christiono et al., 2022^52^	Examination of the effects of administration of saltwater fish powder meal on production of FABP in trophoblast cells and type 1 collagen in ameloblasts	24 pregnant female mice (*Mus musculus*)	Diet supplemented with saltwater fish powder	Control diet without supplementation	IHC	Expression of FABP and type I collagen	Elevated expression of FABP in the mice and diminished expression of type 1 collagen in fetuses in the test group	Maternal diet supplementation alters enamel composition
Gao et al., 2020^40^	Examination of the effects of calcium on ameloblast development and the PI3K/AKT pathway	LS8 cells	Cells treated with various concentrations of Ca^2+^	Cells treated with dimethyl sulfoxide	Flow cytometry, WB	Cell viability, cell morphology, cell cycle, and related regulatory proteins	Calcium inhibits proliferation and promotes differentiation in LS8 cells, downregulation of PI3K/AKT signaling in LS8 cells	Mechanism of Ca^2+^ in tooth mineralization,
Nurbaeva et al., 2018^19^	Demonstration of how calcium enters enamel cells via a transport mechanism involving AcH, ATP, and CCK	Rat secretory and growing enamel organ cells	Cells exposed to Ringers’ solution with Ca^2+^; AcH; ATP or CCK	Cells exposed to only Ringer's solution	IF, Ca^2+^ imaging, RT-PCR, in situ hybridization	SOCE in single ameloblasts, analysis of SOCE regulators CCK, ATP, and AcH	The physiological agonists CCK, AcH, and ATP all stimulate CRAC channel mediated Ca^2+^ entry in ameloblasts	Understanding enamel cell physiology in Ca^2+^ homeostasis, auto/paracrine system for Ca^2+^ transport

AcH, acetylcholine; ALC, ameloblast lineage cells; cCa^2+^, cytosolic Ca^2+^; ER, endoplasmic reticulum; FABP, fatty acid binding proteins; IF, immunofluorescence; IHC, immunohistochemistry; mCa^2+^, mitochondrial Ca^2+^; MCU, mitochondrial Ca^2+^uniporter; RT-qPCR, real time quantitative polymerase chain reaction; SOCE, store operated Ca^2+^ entry; WB, western blot.

Most included studies were performed to examine Ca^2+^ involvement in ameloblast formation through the SOCE pathway (five articles) and STIM pathway (three articles). Another study was performed to examine F⁻ and K^+^ exchange (Table 3).

**Table 3:** Roles of calcium in tooth growth and development.

Tooth developmental stage	Organelle	Role of Ca^2+^	Signaling pathway/molecules
Proliferation	Cell cycle (nuclei)	Stimulated S-phase	Downregulated cyclins A and B, and upregulated cyclin D^40^
Differentiation			Decreased PI3K/AKT/FOXO3, and increased KLK4 and amelotin^40^
	ER, mitochondria	STIM1, SOCE	BMAL1, PER2, TGF-β1, and MAPK14^5^
	Mitochondria	OXPHOS, glycolysis	Decreased LDL and increased PDH in secretory ameloblast stage, ZO-1, and ALP^21^
Maturation	ER, mitochondria	TRMP7 channel, SOCE	Orai-dependent Ca^2+2^
	ER	STIM1, SOCE	Decreased WDR72, STIM1, and ORAIL1, and increased SLC24A4, and CLND2 and 19 under hypoxia^21^
	ER	STIM	Increased KLK4 and CLDN8, and decreased CLDN 1,4 and TJP1/ZO-1^11^
	ER, nuclei	F- exposure	KLK4 decreased through increased expression GRP78 via PERK, elF2α, ATF4, and CHOP^4^
	ER	K^+^ channel	Upregulated Kir42/kcnj15, Slc24a4/Nckx4, Kcnn42, and Kcnh1^65^
	Vesicles, microtubules	K^+^ channel	WDR72^65^
	ER, mitochondria	SOCE, OXPHOS	Increased NCLX/Slc8b1 and mitochondrial Ca^2+^ uniporter (MCU)/mcur1^16^
	ER	SOCE	Increased CCK, ATP, and AcH^19^

## Discussion

Tooth calcification is a mechanism inseparable from Ca^2+^ metabolism. Poor nutrition at this stage significantly induces abnormal tooth size and eruption timing, dental mineralization impairment, caries risk, and disruption of salivary gland function.^34^ Enamel secreted by ameloblasts is the most heavily mineralized tissue in humans^12^^,^^35^ and is composed primarily of substituted hydroxyapatite, which consists predominantly of Ca^2+^ and inorganic phosphate.^36^ The enamel is damaged or partially lost in more than 90% of adult humans, and it cannot be regenerated, because of the lack of ameloblasts in erupted teeth.^12^

Ca^2+^ is essential for signaling in various physiological processes, including the control of circadian rhythms. Ca^2+^ deficiency disorders disrupt the delicate balance in the body, thereby interfering with the circadian rhythm of ameloblasts and impeding enamel synthesis.^1^ An understanding of the complex link between Ca^2+^ deficiency diseases and the molecular circadian clock in ameloblasts is essential to decipher the intricacies of enamel development.^1^ In recent years, significant advances have been made in understanding of the molecular mechanisms underlying enamel formation and the role of ameloblasts. Enhanced knowledge of calcium regulation in enamel development may enable dental professionals to develop advanced preventive care plans and therapies. These methods may aid in preventing enamel defects, decreasing fluorosis risk, and enhancing the teeth's natural regeneration ability. Herein, we present insights into calcium regulation, particularly in ameloblast proliferation, differentiation, and maturation; these findings might play critical roles in the treatment of enamel defects. In addition, we offer insights into future research directions, particularly regarding the molecular pathways with significant potential to enhance understanding and treatment of enamel formation disorders.

A. Roles of calcium in ameloblast proliferation

**Figure 2:**
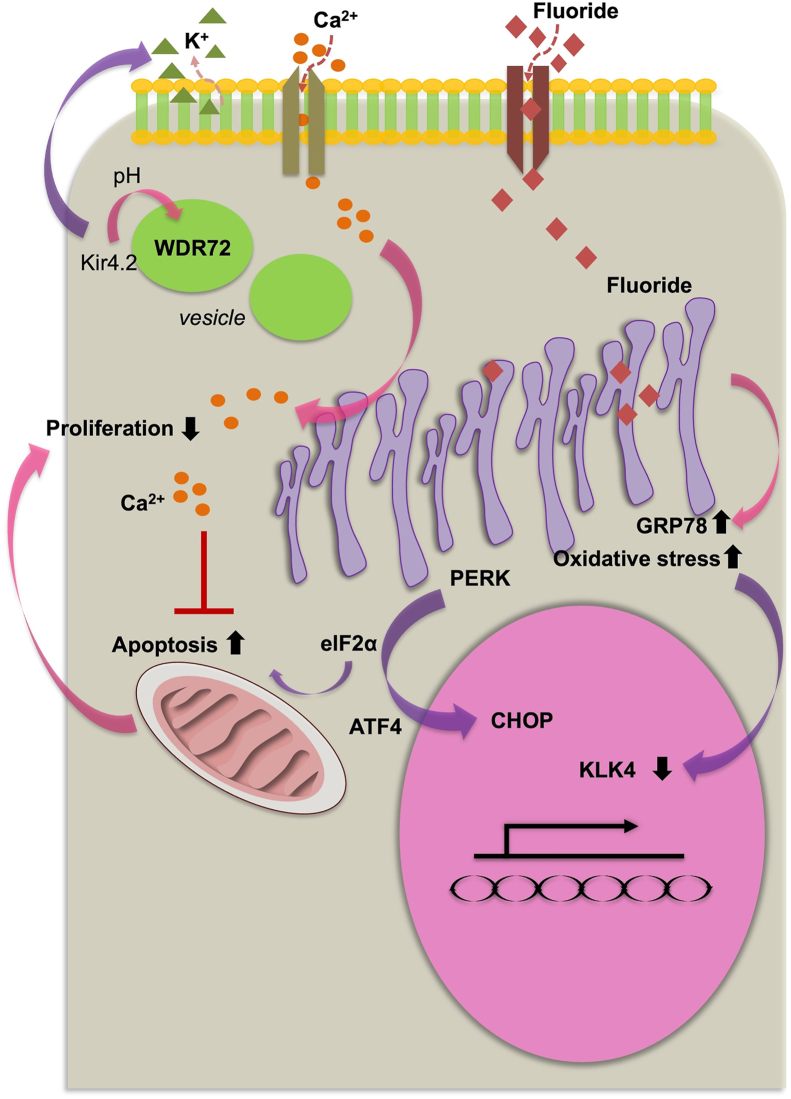
**Calcium and fluoride regulation in ameloblast development**. The elucidation of links among Ca^2+^, fluoride, and ameloblast activity enriches understanding of amelogenesis. Ca^2+^ treatment increases cellular proliferation by limiting the effects of GRP78 and the endoplasmic reticulum (ER) stress pathway, which includes PERK/eIF2α/ATF4/CHOP, and also inhibits the expression of KLK4 and the effects of fluoride-induced apoptosis. Kir4.2 removes K^+^ from the outer layer of tooth enamel. This process involves WDR72 transporting vesicles and regulation of the extracellular matrix pH.

Ameloblast proliferation is maintained by intracellular Ca^2+^ homeostasis, which is required or enamel formation.^37^ The inner enamel epithelium, which is of ectodermal origin, proliferates and differentiates into secretory-stage ameloblasts that subsequently mature.^21^^,^^36^ F^−^ and Ca^2+^ levels are known to affect ALCs. The proliferation of these cells increases under exposure to low F^−^ concentrations and decreases significantly under exposure to high (>1 mM) concentrations, with the induction of apoptosis and disruption of the cell cycle.^38^ Combined Ca^2+^ and F^−^ treatment of ALCs disrupts Ca^2+^ homeostasis and significantly decreases intracellular Ca^2+^ levels below those observed with F^−^ treatment alone,. However, Ca^2+^ helps counteract the inhibition of cell proliferation caused by F^−^, as evidenced by enhanced expression of KLK4 in ALCs.^4^ F^−^ has been suggested to disrupt inositol trisphosphate (IP3) receptors, mitochondrial respiration, and cellular bioenergetics, as reflected by ultrastructural changes in the endoplasmic reticulum (ER) and elevated expression of the ER stress marker glucose-regulated protein 78 under F^−^ treatment.^4^ This ER stress activates the protein kinase R-like ER kinase and the α subunit of eukaryotic initiation factor 2, which in turn activates transcription factor 4 and the CCAAT enhancer-binding protein homologous protein pathway (Figure 2). Ca^2+^ supplementation counteracts the harmful effects of F^−^ in ALCs, and might decrease fluorosis. Ameloblast apoptosis might be induced by excessive F^−^ and mitigated by Ca^2+^. The disruption of Ca^2+^ homeostasis and inhibition of cell proliferation are closely associated with apoptosis.^4^

Disruptions in the cell cycle accompany variations in cell proliferation.^39^ F^−^ has been shown to halt ALC growth for 24 h in the G0/G1 phase and for 48 h in the G2/M phase. G0/G1 cell cycle arrest prevents injured cells from proceeding with DNA replication during this phase, and is accompanied by the suppression of cell division and the initiation of programmed cell death.^4^^,^^39^ Gao et al.^40^ have found that comparable quantities of Ca^2+^ exert opposite effects from F^−^, by decreasing the durations of the G0/G1 and G2/M phases in the ameloblast-like LS8 cell line; these findings are attributable to the effects of F^−^ on Ca^2+^ and various cellular phenotypes.

Jedeon et al.^41^ have analyzed ameloblast proliferation in the HAT-7 rat dental epithelial cell line. Ameloblasts express estrogen receptors, which enhance their proliferation and transcription. Treatment of HAT-7 cells with the estrogen receptor antagonist ICI 182, 780 has been found to inactivate estrogen receptors α and abolish the effects of estradiol on ameloblast proliferation and transcription, but to only partially mitigate the effects of bisphenol A.^42^ BPA affects amelogenesis in male rats more prominently, exerting both ER-dependent and ER-independent effects on ameloblast proliferation and gene transcription.^42^ The estrogen signaling pathway is involved in tooth development and enamel mineralization.^41^ However, the crosstalk between Ca^2+^ and hormonal regulation during ameloblast proliferation remains unclear.

B. Influence of calcium on ameloblast differentiation

Most investigations of tooth formation and molecular analysis have been performed in mouse models. However, the growth and development mechanisms of mouse and human teeth differ. For instance, mouse incisors regenerate continually throughout life, owing to the presence of epithelial stem cells in the labial cervical loop, thereby enabling ongoing enamel creation. The understanding of tooth differentiation during early human development is important, because no such regeneration mechanism occurs in adult human teeth.^42^ The formation of human oral tissue begins approximately 6 weeks after conception, with the thickening of the oral epithelium. This thickening gives rise to all primary teeth, which develop as extensions of the main dental lamina. The teeth undergo a series of morphological stages (bud, cap, and bell) in bony crypts in the jaws.^43^

Teeth develop as outgrowths of the outer (ectodermal) cell layer; their formation is controlled by connections between different tissues, which are facilitated by networks of signaling pathways that are conserved across species.^44^ A developing tooth contains a compartment called the enamel organ, comprising inner and outer enamel epithelial cells, among others.^6^^,^^45^ The inner enamel epithelial cells differentiate into ameloblasts, which progress through presecretory, secretory, transitional, and maturation stages. Enamel formation is initiated in the presecretory stage, during which secretory ameloblasts deposit an organic protein-enriched matrix onto the dentin. During this initial phase, specificity protein (Sp)6 is involved in the determination of cell fates. Sp6-driven ameloblast differentiation culminates in maturation and enhances the ability to regulate enamel mineralization via the expression of amelotin and KLK4.^46^ During the secretory stage, specific tall columnar ameloblasts release proteins including amelogenin, ameloblastin, and enamelin, which form enamel.^36^

**Figure 3:**
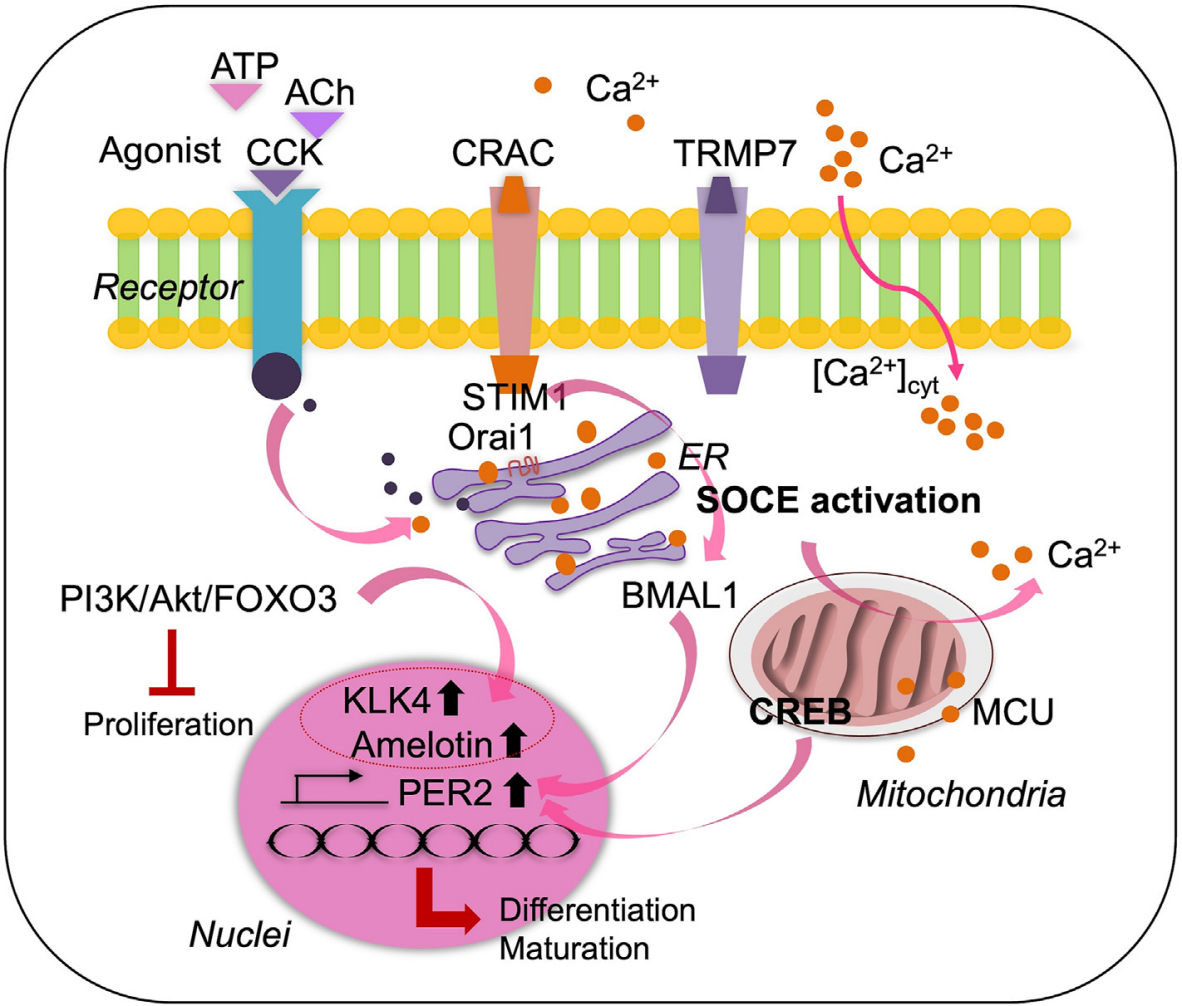
**Proposed model of signal-mediated and signal-activated Ca**^**2+**^**entry into ameloblasts**. CCK, Ca^2+^, ATP, and ACH treatments increase Ca^2+^ in the cytoplasm; this process is mediated by CRACs through the activation of SOCE. The TRPM7 channel is a potential modulator of ORAI-dependent Ca^2+^ uptake. Phosphorylated CREB in the mitochondria translocates to the nucleus, where it binds the cAMP response element (CRE) of the PER2 gene promoter, thereby inducing its expression through the mitochondrial calcium uniporter (MCU) pathway. Simultaneously, the brain and muscle ARNT-like protein 1 (BMAL1) heterodimerizes in the cytoplasm and also translocates to the nucleus, where it enhances PER2 expression and acts as a key regulator of its own repressor. The coordinated actions of CREB and BMAL1 in the nucleus contribute to the precise regulation of PER2 expression. Ca^2+^ treatment decreases PI3K/AKT/FOXO3 expression, thereby inhibiting ameloblast proliferation and increasing the expression of KLK4 and amelotin, which are markers of the ameloblast maturation stage.

Ameloblast differentiation is hindered at the molecular level by the abnormal accumulation and activation of nuclear factor erythroid 2–related factor 2, a specific target of autophagy.^47^ During the presecretory stage of amelogenesis, the transcription factor activator protein (AP)-2α is highly expressed and facilitates the transition of pre-ameloblasts into secretory ameloblasts.^48^ The activity of the phosphatidylinositol 3-kinase (PI3K)-Akt signaling pathway during tooth germ differentiation has been found to play important roles in the induction of amelogenin, ameloblastin, and enamelin expression and calcification in a mouse dental epithelial cell line.^49^ The TRPM7 channel is also abundantly expressed in ameloblasts, where it regulates cellular levels of Ca^2+^, zinc ions, and magnesium ions (Mg^2+^).^2^ The kinase domain of TRPM7 is particularly important in the early stages of ameloblast differentiation, because it activates bone morphogenetic protein signaling by phosphorylating the cyclic adenosine monophosphate (cAMP) response element–binding protein, thereby supporting the initiation of ameloblast maturation (Figure 3).^50^

Ca^2+^ suppresses cell proliferation and enhances differentiation by decreasing PI3K/AKT signaling activity. Recent findings have indicated that Ca^2+^ inhibits LS8 cell proliferation while promoting differentiation through the PI3K/Akt pathway. Treatment of these cells with Ca^2+^ concentrations ranging from 2.0 to 3.5 mM has been found to slightly decrease cell survival accompanied by cell cycle arrest in the S phase, and to change the levels of cyclins A, B, and D, and upregulate differentiation markers such as KLK4 and amelotin. Western blot analysis has confirmed decreasing PI3K, Akt, phosphorylated AKT, and forkhead box O3 expression with exposure to increasing Ca^2+^ concentrations in LS8 cells. These findings suggest that Ca^2+^ plays a critical role in the modulation of the PI3K/Akt pathway, thereby facilitating LS8 cell differentiation, similarly to its role in ameloblast maturation.^40^

C. Calcium-dependent pathways in ameloblast maturation

Maturation-stage ameloblasts facilitate the movement of Ca^2+^ from the bloodstream to mineralization areas on apical cell surfaces. These cells also regulate the acidity of the enamel formation region by altering the extracellular pH (6.2–7.2) and switching between ruffle-ended and smooth-ended forms. Thus, ameloblasts are affected by small variations in pH, which in turn affect Ca^2+^ absorption and release.^6^^,^^26^^,^^51^ Collagen expression decreases from the secretory phase to the mineralization and maturation phases of ameloblasts, and further decreases in the enamel matrix until the post-metamorphic juvenile dentition phase-field during amelogenesis.^52^ The ameloblast maturation phase has been investigated in numerous analyses (Table 3). We provide a comprehensive discussion of the multiple pathways involved in this phase.

### The SOCE pathway

SOCE supplies ameloblasts with Ca^2+^, which is essential for enamel crystal formation.^18^^,^^19^ These crystals possess enhanced Ca^2+^ transport ability, owing to increased Ca^2+^ uptake facilitated primarily by SOCE.^19^^,^^53^ SOCE significantly increases cytosolic Ca^2+^ (cCa^2+^) levels in maturation-stage rat ameloblasts compared with secretory ameloblasts.^54^^,^^16^

Stimulation of Ca^2+^ entry via SOCE and inhibition of the mitochondrial Ca^2+^ uniporter (MCU) with the inhibitor Ru265 hinder cCa^2+^ clearance in permeabilized enamel (LS8) cells (ruthenium red has a similar effect) but have no effect on the mitochondrial membrane potential of intact cells. SOCE stimulation enhances the absorption of mitochondrial Ca^2+^ (mCa^2+^) in maturation-stage ameloblasts, as compared with primary ameloblasts.^16^ Ameloblast maturation involves the regulation of mCa^2+^ via SOCE.^16^ The activation of SOCE markedly increases mCa^2+^ absorption by maturing ameloblast mitochondria, a process mediated by MCU.^16^ The loading of secretory and maturing cells with the non-ratiometric markers fluo4AM and rhod2AM enable the simultaneous quantification of cCa^2+^ and mCa^2+^ uptake.^16^^,^^55^

Increased expression of STIM1, STIM2, TRPC1, and ORAI1 in rat enamel organs during maturation provides additional evidence of the involvement of SOCE in primary ameloblast maturation.^16^^,^^54^^,^^56^ Ca^2+^ might promote the differentiation of certain LS8 cells from the secretory stage to the maturation stage, given these cells’ diminished survival rates, and the elevated expression of amelotin and KLK4. This hypothesis is partly consistent with a prior finding that Ca^2+^ is involved in the induction of primary human ameloblast precursor differentiation.^40^^,^^57^

The Na/Li/Ca exchanger (NCLX) is the primary transporter responsible for the expulsion of Ca^2+^ from the mitochondria.^58^ The expression s of Slc8b1, which encodes NCLX, increases approximately two-fold during the maturation of enamel cells stimulated with adenosine triphosphate (ATP) in the presence of the NCLX inhibitor CGP-37157. NCLX blockade inhibits the release of mCa^2+^, as evidenced by an increase in rhod2AM fluorescence in secretory- and maturation-stage cells.^58^ In maturing cells, mCa^2+^ retention is substantially elevated, thus indicating more pronounced NCLX activation.^16^

Cholecystokinin (CCK), a potential SOCE activator, is associated with the presence of CCK receptor transcripts. Ca^2+^ imaging has revealed that stimulation with CCK increases the concentration of Ca^2+^ in the cytoplasm in a dose-dependent manner, whereas this effect is inhibited by calcium release–activated channel (CRAC) inhibitors. Acetylcholine and ATP, whose receptors are present on enamel cells, also activate SOCE and exert comparable effects.^19^ These findings provide initial evidence of a potential SOCE regulatory mechanism in enamel cells, thus reinforcing the concept of Ca^2+^ transcytosis in the ER as a means of transporting large amounts of Ca^2+ 19^. SOCE is activated by the release or depletion of Ca^2+^ from intracellular stores. This process can occur via the activation of IP3 receptors or the inhibition of sarcoplasmic/ER Ca-ATPase. The depletion of Ca^2+^ reserves from the ER causes the accumulation of STIM at the ER–plasma membrane junction.^16^

### The STIM pathway

STIM1, early growth response (EGR) protein 1, and nuclear receptor subfamily 2 group F member 6 (NR2F6) are highly expressed in the ameloblast maturation stage.^5^^,^^59^^,^^60^ EGR1 is a major regulator of STIM1, and its expression is induced by transforming growth factor-beta 1 (TGF-β1) in maturation-stage ameloblasts.^59^^,^^61^ TGF-β1 and STIM1 play important roles in the regulation of ameloblast function during enamel maturation, by interacting with KLK4 and matrix metalloprotease 20.^62^ STIM1 targeting significantly alters expression of TGF-β1 and several other circadian regulators, including p38α and mitogen-activated protein kinase (MAPK) 14.^5^ STIM1 deletion upregulates the Bmal1 gene and downregulates the Per2 circadian gene.^5^ MAPK14 in ameloblasts is involved in regulating early tooth morphogenesis, whereas its deletion in the ectodermal tissue results in the formation of irregularly shaped dental cusps and considerable underdevelopment of the enamel layer.^63^

MAPK14 expression is markedly elevated in ameloblasts lacking STIM1. MAPK14 modulates SOCE via indirect mechanisms involving TGF-β1 and nuclear factor-kappa B (NF κB), as well as via direct phosphorylation of STIM1.^5^ The observed differential regulation of circadian clock–related genes in STIM1 cKO mouse teeth—with the downregulation of TGF-β1, EGR1, and NR2F6 in the maturation stage, and the upregulation of MAPK14 and transcription factor AP-2α in the presecretory and secretory stages—provides evidence of the intricate downstream effects of altered SOCE signaling in ameloblasts.^5^

Maturation-stage ameloblasts are responsible for terminal enamel mineralization, and undergo characteristic cyclic morphological and functional alterations between ruffle-ended and smooth-ended forms.^21^ Hypoxia significantly decreases the expression of mRNAs associated with transcellular Ca^2+^ transport, such as WDR72, STIM1, and ORAI1, and increases expression of *SLC24A4*, the gene encoding Na^+^/K^+^/Ca^2+^ exchanger 4 (NCKX4).^9^^,^^21^ Mutations in WDR72 decrease the quantity and dimensions of blood vessels in the capillary layer and affect the subcellular positioning of SLC24A4, a protein with a crucial role in the transcellular transfer of Ca^2+^ in maturation-stage ameloblasts.^64^ However, hypoxia decreases the transport of Ca^2+^ across HAT-7 cells, and increases the expression of mRNAs for claudins 2 and 19; therefore, these molecules appear to be involved in blocking such transport during ameloblast maturation.^21^

During enamel maturation, the levels of Na^+^ and K^+^ in the matrix decline gradually.^65^ Ameloblast modulation involves the movement of NCKX4 to the apical edges of ruffle-ended ameloblasts, thereby enabling removal of Na^+^ from the enamel matrix and exchange for Ca^2+^ and K^+^.^65^ The levels of Na^+^ and K^+^ in mature enamel are lower in normal mice than fluorotic mice,^66^ because of impaired NCKX4 transport in fluorosed ameloblasts.^66^^,^^67^

SLC24A4 is significantly upregulated during enamel maturation, with respect to its expression in secretory enamel organs. Expression of Kcnn4 and Kcnh1 is greater throughout the maturation stage than the secretory stage. Kcnj15 (Kir4.2) is synthesized by maturation-stage enamel organs and is localized to the ameloblast apical border.^65^ Kir4.2 participates in K^+^ uptake by maturing ameloblasts, and K^+^ and Na^+^ uptake by Kir4.2 and Nckx4, respectively. These processes might be regulated by pH via WDR72-mediated endocytosis and membrane trafficking.^65^ In fluorosed and Wdr72^−/−^ mice, translocation of NCKX4 to the apical membrane is diminished, and Kir4.2 is found predominantly in the cytoplasm.^64^^,^^66^ Purinergic G protein–coupled receptors regulate the activity of the NCKX4 Ca^2+^ extrusion pathway, which plays a critical role in dental enamel maturation.^53^^,^^68^

The expression of WDR72, which is indispensable for microtubule assembly and vesicular transport in maturation-stage ameloblasts, has been reported to be upregulated in LS8 cells at an acidic pH of 6.2, corresponding to the acidity of the enamel matrix beneath ruffle-ended ameloblasts.^65^ Kir4.2 is responsible for removing K^+^ from the outer layer of the tooth enamel—a process associated with the transport of vesicles by WDR72 and the regulation of pH in the extracellular matrix.^65^^,^^69^

A novel 3D model constructed with HAT-7 cells has been used to study the intricate molecular mechanisms of amelogenesis, thus enabling the examination of enamel formation and enamel disorders resulting from F^−^ exposure. This model has been used to study the effects of external stimuli on intracellular Ca^2+^ signaling, pH regulation, and ultimately enamel mineralization.^11^ The most notable change observed is in the expression of KLK4, thus suggesting a shift toward the maturing ameloblast phenotype during 3D organization.^11^^,^^70^ KLK4 expression has been found to be approximately 70 times higher in a 3D culture of HAT-7 spheroids than in a two-dimensional monolayer culture.^11^

KLK4 and amelotin are secreted throughout the transition and maturation periods, respectively. The primary role of KLK4 is the degradation of enamel matrix proteins. Amelotin, a member of the secretory Ca^2+^-binding phosphoprotein family, participates in enamel mineralization and ameloblast attachment to the enamel during maturation. A mutation in the KLK4 gene can lead to the production of protein residues in the enamel, thus making the enamel porous and soft.^71^ Levels of the tight-junction protein (TJPs) claudin-1, claudin-4, and TJP1/zonula occludens 1 are significantly diminished, whereas that of claudin-8 is elevated, in 3D HAT-7 culture.^11^ Moreover, the expression of SLC26A4/pendrin and cystic fibrosis transmembrane conductance regulator (CFTR) is significantly diminished in spheroids.^21^ These proteins are typically found in the apical membranes of ameloblasts. CFTR is highly expressed in maturation-stage ameloblasts, whereas its expression is weak in the transition stage and negligible in the secretory stage.^67^ The expression levels of the basolateral transporter sodium/proton exchanger 1, anion exchange protein 2, and electrogenic sodium bicarbonate cotransporter are slightly, but not significantly, lower in two-dimensional HAT-7 culture than in HAT-7 spheroids.^11^

### The TRMP7 pathway

TRPM7 channels are essential for Ca^2+^ transport during amelogenesis,^2^ and the fatty acid–binding protein (FABP) and type 1 collagen expression levels influence enamel quality. Furthermore, the observed changes in the physical characteristics of ameloblasts under low-oxygen conditions highlight the significance of energy metabolism in enamel development and maturation.^52^ Kádár et al.^2^ have documented TRPM7's direct regulation of Ca^2+^ transport across epithelial cells during amelogenesis. TRPM7 might regulate ORAI-dependent Ca^2+^ uptake and function as a separate Ca^2+^ entry mechanism that is affected by pH in HAT-7 cells.^2^ The considerable effects of Ca^2+^ and circadian rhythms on ameloblasts can aid in deciphering the mechanistic links between intracellular Ca^2+^ dynamics and the molecular circadian clock.^5^ cAMP and Ca^2+^ signaling have been documented to contribute to the regulation of cellular timekeeping, and to be regulated by the cellular clock.^72^

The interactions of clock genes (including circadian locomotor output cycles kaput, brain muscle aryl hydrocarbon receptor nuclear translocation, period 1–3, cryptochrome 1 and 2, Cry2, and SOCE) form perpetual autoregulatory transcription-translation feedback loops that control the rhythmic expression of these genes over 24-h cycles, to achieve normal functioning.^73^ cAMP and Ca^2+^ directly affect these feedback loops.^74^ Transcellular Ca^2+^ transport in ameloblasts occurs via high-capacity intracellular stores in the ER (SOCE), and is mediated primarily by the ER transmembrane proteins STIM1 and STIM2, and highly selective plasma membrane CRACs.^13^^,^^53^^,^^75^ STIM1 and STIM2 serve as intracellular Ca^2+^ sensors, whereas transmembrane CRAC proteins (ORAIs) 1–3 form pores in CRACs and serve as filters during Ca^2+^ entry into ameloblasts from the circulation.^13^^,^^76^

### Directions for future research on calcium signaling and enamel formation pathways

Research on the TRPM7 and SOCE pathways has provided valuable insights into calcium signaling in ameloblasts, as well as the effects of these mechanisms on enamel formation and integrity.^77^ Currently, no direct evidence indicates that TRPM7 physically interacts with CRACs (STIM1/ORAI1 channels) in ameloblasts or other cell types. These calcium channels are separate but play complementary roles in the regulation of calcium influx during amelogenesis.^77^^,^^78^ TRPM7 mediates direct Ca^2+^ and Mg^2+^ transport into ameloblasts, whereas CRACs respond to depleted intracellular Ca^2+^ stores. Both systems are crucial for the maintenance of the high levels of calcium required for proper enamel mineralization, and might work synergistically in maintaining overall calcium homeostasis, with TRPM7 contributing to basal calcium levels and CRACs ensuring replenishment when calcium stores are low. Better understanding of these molecular mechanisms might shed light on enamel defects and indicate future research directions for dental treatments. For example, analysis of the crosstalk between TRPM7 and SOCE and its influence on the Ca^2+^ signaling cascade might provide new insights.^77^

### Optimization of calcium–fluoride interaction for targeted intervention and treatment of enamel defects

The targeted treatment of developmental enamel defects, such as hypomineralization and hypoplasia, has increasingly used calcium-modulating agents. These agents promote remineralization and aid in the repair of mild enamel defects, thus offering a non-invasive treatment strategy to enhance natural repair processes. When enamel damage is detected early, such interventions can help restore enamel strength and decrease susceptibility to caries. In parallel, a deeper understanding of calcium dynamics in the context of enamel development has important implications for refining fluoride dosing strategies.^79^ Fluoride remains a cornerstone of caries prevention, as reflected by community water fluoridation programs and individual-use products, such as toothpaste and supplements.^80^^,^^81^ Optimization of the balance of calcium–fluoride interaction might minimize fluorosis risk while retaining the protective effects of fluoride against caries.^82^

Calcium–fluoride interaction has emerged as a key focus for the prevention of enamel under- and over-mineralization. In cases of excessive fluoride exposure, strategies for modulating calcium levels might be explored to correct fluorosis or improve enamel quality by regulating calcium deposition during enamel maturation.^80^^,^^83^ Specifically, fluoride triggers the unfolded protein response, a cellular stress response that impairs calcium signaling and transport.^83^ This response decreases the availability of calcium during maturation and results in hypomineralized, porous enamel characteristic of dental fluorosis. Fluoride inhibits SOCE in ameloblasts, thus further decreasing calcium entry and exacerbating enamel defects.^83^

Calcium's role extends beyond passive remineralization, and is also crucial for the differentiation of dental stem cells into ameloblast-like cells, which might be critical for enamel regeneration in patients with congenital or acquired enamel defects.^84^ Ameloblast differentiation is calcium dependent, and the optimization of calcium levels in regenerative therapies might improve functional enamel formation.^85^ Preventive interventions implemented during critical periods of tooth development, particularly in utero, are essential for promoting healthy enamel formation.^50^ Ensuring optimal calcium intake during tooth development is crucial, because disruptions in calcium availability or regulation can impair enamel mineralization and lead to defects such as hypomineralization.^85^

The interaction between calcium and fluoride during enamel formation is crucial in the development of preventive and therapeutic strategies. Optimizing calcium intake and refining fluoride dosing can support enamel health and decrease the risk of fluorosis. Calcium's roles in ameloblast differentiation and enamel regeneration also have potential in treating enamel defects and restoring enamel integrity.

### Insights into clinical applications of calcium regulation for enamel defects

Herein, a molecular mechanism to understand the physiologic and pathologic changes due to calcium transport during amelogenesis is described (Table 2), on the basis of data primarily from experimental preclinical studies in cells or animals. Clinical translational protocols to understand the molecular changes occurring in developmental stages remain lacking. In terms of clinical relevance, calcium transport affects tooth mineralization either during the formative stage, by altering the maternal or infant diet, thus directly affecting amelogenesis, or after enamel formation, with extrinsic substances such as mouthwashes or varnishes.^52^ Recent trends in of prenatal and postnatal dietary supplementation, and their overall effects on bone and oral health, have been extensively reported.

Vitamin D is crucial for calcium metabolism and mineralization process, and several studies have reported prenatal vitamin D deficiency and defects in enamel.^86^ Prenatal diet and supplementation influence the bone and dental health of the offspring. Strategies to achieve the full benefits of vitamin D supplementation in the maternal diet have been shown to affect enamel defects in primary teeth and permanent molars.^87^ No differences in the prevalence of early childhood caries (ECC), which may originate prenatally, have been observed between prenatal vitamin D supplemented and control groups.^88^ Vitamin D supplementation in infants has been found to be more effective in controlling ECC and severe-ECC than maternal prenatal supplementation.^89^ Protective effects of vitamin D against hypomineralized second primary molars and molar incisor hypomineralization have also been studied.^90^ Investigation of reverse translation of all observed clinical findings to determine effects on the calcium transport mechanism might prove valuable in developing more effective dietary supplementation guidelines for healthy enamel formation.

### Strengths and limitations

The strength of this literature review lies in its comprehensiveness, including tooth development and formation processes including enamel formation and Ca^2+^ regulation. Most of the included articles explained the major roles of Ca^2+^ in enamel and tooth formation, and highlighted the importance of methodological standards for experimental design, data collection, and analysis, to ensure reliability and reproducibility of findings in investigations of Ca^2+^ regulation and enamel formation. However, the inherent limitations of scoping reviews must be acknowledged. Potential bias regarding the role of Ca^2+^ in ameloblasts in tooth formation might have been introduced, because only published scientific studies were considered. Additionally, the stipulated date and language restrictions might have affected the accuracy of the research, and resulted in the exclusion of certain topics. Moreover, all included studies were found to have moderate-to low-quality study designs, thus indicating the need for more robust and standardized protocols for preclinical research.

## Conclusion

This thorough examination of factors influencing ameloblasts and enamel formation highlights the intricate interplay among molecular, environmental, and dietary factors. Ca^2+^ deficiency, F^−^ exposure, and the roles of various channels and proteins, such as TRPM7, FABP, and Kir4.2, have been investigated in various experimental settings. The use of 3D models, and the examination of gene expression patterns and the influence of energy metabolism on ameloblast maturation, has contributed to in-depth understanding of enamel development. In addition, research has underscored the potential of Ca^2+^ supplementation to mitigate the toxic effects of F^−^ and aided in the exploration of the regulatory mechanisms involved in Ca^2+^ transport and transcytosis in ameloblasts. Overall, these novel findings have contributed to the evolving landscape of dental research, by opening avenues for further investigation and potential development of therapeutic interventions.

## Source of funding

No specific grant was awarded for this study.

## Ethical approval

The authors declare no ethical concerns that require disclosure.

## Authors contributions

IRH contributed to the conception design of the study, analyzed the results, and drafted the article. RCD and AR conducted the systematic review. DYA, IRH, and SA significantly contributed to critical revision, quality assessment, and improving the article's language and style. YSR and SC revised the manuscript and approved the final version. Each author has thoroughly examined and approved the final version of the article, and is responsible for the content and similarity index. All authors have critically reviewed and approved the final draft and are responsible for the content and similarity index of the manuscript.

## Conflict of interest

The authors declare no conflicts of interest.
